# Genome assembly and annotation report of *Arthrobacter* sp. SA17, a potential new species isolated from saffron (*Crocus sativus* L.) rhizosphere in Morocco

**DOI:** 10.1128/mra.01454-25

**Published:** 2026-03-31

**Authors:** Rahma Zouagui, Houda Zouagui, Laila Sbabou

**Affiliations:** 1 Microbiology and Molecular Biology Team, Center of Plant and Microbial Biotechnology, Biodiversity and Environment, Faculty of Science, Mohammed V University in Rabat, Rabat, Morocco; Indiana University, Bloomington, Bloomington, Indiana, USA

**Keywords:** *Arthrobacter *sp., *Crocus sativus*, rhizosphere, draft genome sequence, genome annotation

## Abstract

This report presents the draft genome sequence and annotation features of *Arthrobacter* sp. SA17, a rhizobacterium isolated from saffron rhizosphere in Morocco. The genome harbors genes involved in auxin and siderophore biosynthesis, inorganic phosphate solubilization, and polyamine metabolism and transport.

## ANNOUNCEMENT

The rhizosphere, the soil surrounding plant roots, hosts microbial communities involved in nutrient solubilization, phytohormone production, and plant stress tolerance (1, 2). *Arthrobacter* sp. SA17 was isolated from the rhizosphere of saffron (*Crocus sativus* L.) from a farm in Taliouine-Taznakht, Morocco (30°28′13″N, 7°46′22″W). Soil samples were suspended in sterile 0.9% (wt/vol) NaCl, serially diluted in six 10-fold steps (10⁻¹–10⁻⁶), and 100 µL of 10⁻³–10⁻⁵ dilutions was plated, following Becerra-Castro et al. (3). Aliquots from each selected dilution were plated on three screening media: Pikovskaya’s for phosphate solubilization (4), Modi for siderophore production (5), and yeast extract-mannitol (YEM)-tryptophan for auxin production (6). Plates were incubated at 28°C for 48 h, and colonies were purified by four streaks. *Arthrobacter* sp. SA17 was isolated on Pikovskaya’s medium and showed a clear halo, indicating phosphate solubilization. The strain also produced siderophores on Modi medium, indicated by a colored halo, and auxin on YEM medium, detected by a color change after addition of Salkowski reagent.

Genomic DNA was extracted from a single colony grown in Luria-Bertani broth (7) at 28°C for 24 h using the QIAamp Genomic DNA Mini Kit (Qiagen, Germany). DNA purity and concentration were assessed with a Nanodrop 2000 and Qubit 3.0, and integrity was checked by electrophoresis on a 0.8% agarose gel. The sequencing library was prepared with the Rapid Barcoding Sequencing Kit (SQK-RBK004) and sequenced on a MinION MK1C device (Oxford Nanopore Technologies, UK) using an R9 flow cell for 72 h. The reads were basecalled and demultiplexed using Guppy v5.0.7 (8), trimmed with Porechop v0.2.4 using default parameters (9), *de novo* assembled with Canu v2.2 (10), and polished with three rounds of Racon v1.5.0 (11) followed by Homopolish v0.3.4 against 562 *Arthrobacter* genomes (12). The assembled contigs were annotated using the Rapid Annotation using Subsystems Technology server (13) and NCBI Prokaryotic Genome Annotation Pipeline v6.2 (14). Biosynthetic gene clusters of secondary metabolites were predicted with antiSMASH (15). Strain identification was performed using the Type Strain Genome Server (16) and confirmed by average nucleotide identity (ANI) calculations with pyANI v0.2.9 (17).

The raw sequencing data generated 183,237 reads, with an N50 read length of 4,043 bp and 80.48% of bases having a quality score above 7. The draft genome of *Arthrobacter* sp. SA17 consists of two contigs, totaling 4,816,891 bp with 62% GC content, a contig N50 of 4,808,011 bp, and 85× average coverage. Genome annotation predicted 6,242 coding sequences, 3 rRNA, and 50 tRNA genes. *Arthrobacter* sp. SA17 showed the highest digital DNA–DNA hybridization (dDDH) value of 37.7% and ANI of 89.22% with *Arthrobacter rhizosphaerae* CCNWLXL 1-35^T^ (GCF_023062595.1), both below the species cutoffs of 70% for dDDH (18) and 95% for ANI (19), indicating it represents a novel species.

**Fig 1:**
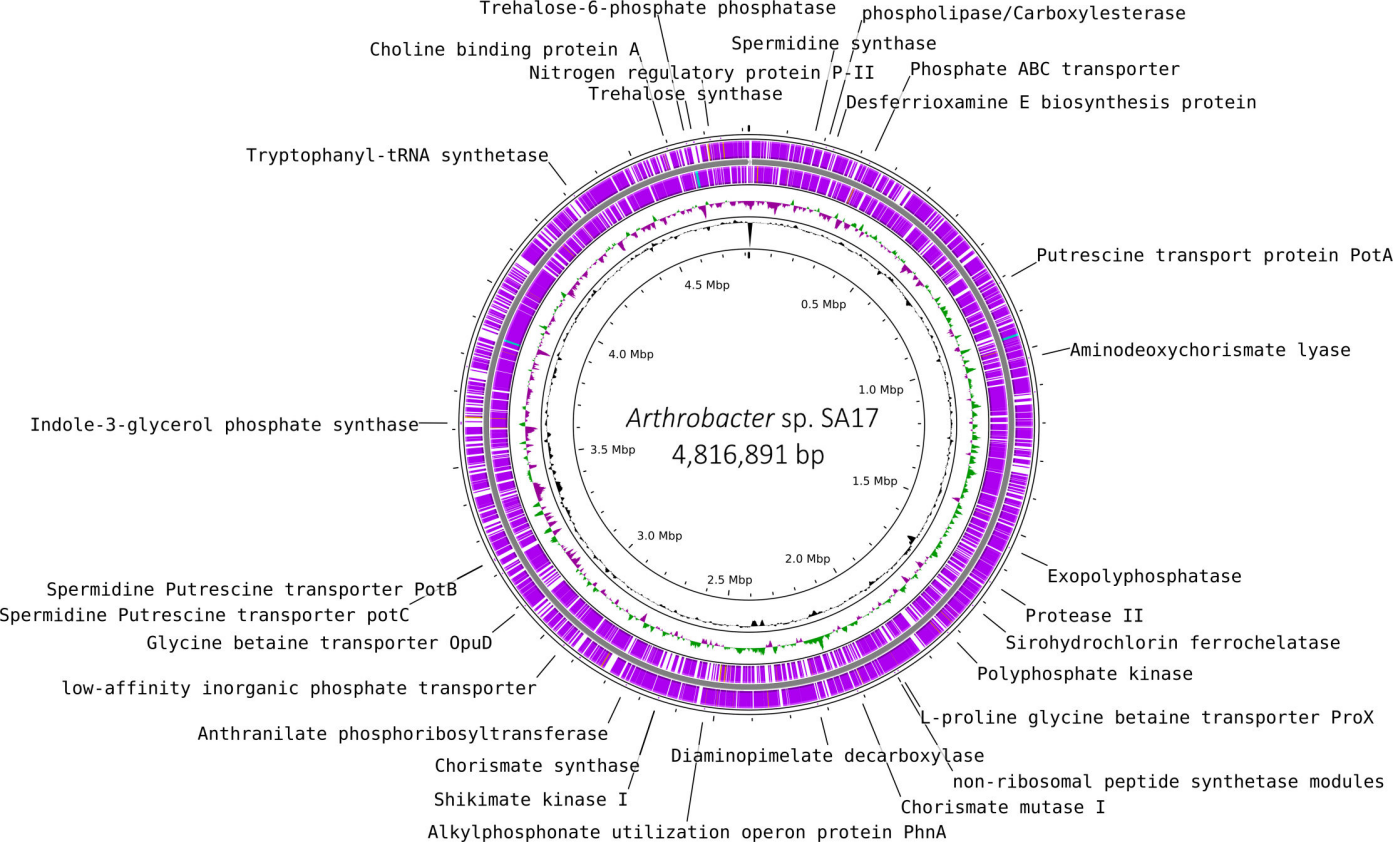
Circular map of the draft genome of *Arthrobacter* sp. SA17. From the outermost ring to the center: the purple rings (rings 1 and 2) show genes on the forward and reverse strands, respectively; ring 3 shows the GC content; and ring 4 shows the GC skew. Genes associated with plant growth-promoting traits are indicated and labeled in black at the edge of the map. The map was generated using PROKSEE (https://proksee.ca/) (20).

The genome of *Arthrobacter* sp. SA17 contains key genes for phosphate solubilization, auxin production, nitrogen metabolism, iron uptake, polyamine transport and metabolism, quorum sensing, and chemotaxis (Fig. 1). Secondary metabolite analysis revealed a high-confidence biosynthetic gene cluster for the siderophore desferrioxamine B.

## Data Availability

The assembled genome of *Arthrobacter* sp. SA17 is deposited in DDBJ/ENA/GenBank under accession GCA_053891455.1. Raw sequencing reads are available in the SRA under SRX29993469, with BioProject PRJNA827450 and BioSample SAMN50023085.
